# Evaluation of Patellar Contact Pressure Changes after Static versus Dynamic Medial Patellofemoral Ligament Reconstructions Using a Finite Element Model

**DOI:** 10.3390/jcm8122093

**Published:** 2019-12-01

**Authors:** Vicente Sanchis-Alfonso, Gerard Ginovart, Diego Alastruey-López, Erik Montesinos-Berry, Joan Carles Monllau, Angel Alberich-Bayarri, María Angeles Pérez

**Affiliations:** 1 Department of Orthopaedic Surgery, Hospital Arnau de Vilanova, Carrer de Sant Clement 12, 46015 Valencia, Spain; 2 Department of Orthopaedic Surgery, Hospital Terres de l’Ebre, 43500 Tortosa, Spain; gerardg@intersoft.net; 3 Multiscale in Mechanical and Biological Engineering (M2BE), Aragón Institute of Engineering Research (I3A), Department of Mechanical Engineering, University of Zaragoza, 50018 Zaragoza, Spain; d.alastruey@gmail.com (D.A.-L.); angeles@unizar.es (M.A.P.); 4 Orthopaedic Surgeon, Agoriaz Orthopaedic Center, Riaz and Clinique CIC, 1832 Montreux, Switzerland; erik.montesinos@gmail.com; 5 Department of Orthopaedic Surgery and Traumatology, Hospital del Mar, Universitat Autònoma de Barcelona, 08003 Barcelona, Spain; jcmonllau@gmail.com; 6 Quantitative Imaging Biomarkers in Medicine (QUIBIM) SL, GIBI230, Biomedical Imaging Research Group, La Fe Health Research Institute, 46026 Valencia, Spain; angel@quibim.com

**Keywords:** MPFL reconstruction, finite element model, patellar contact pressure, patellar cartilage degeneration after MPFL reconstruction

## Abstract

Objectives: To evaluate the effect of various medial patellofemoral ligament (MPFL) fixation techniques on patellar pressure compared with the native knee. Methods: A finite element model of the patellofemoral joint consisting of approximately 30,700 nodes and 22,200 elements was created from computed tomography scans of 24 knees with chronic lateral patellar instability. Patellar contact pressures and maximum MPFL graft stress at five positions of flexion (0°, 30°, 60°, 90°, and 120°) were analyzed in three types of MPFL reconstruction (MPFLr): (1) static/anatomic, (2) dynamic, using the adductor magnus tendon (AMT) as the femoral fixation, and (3) dynamic, using the quadriceps tendon as the attachment (medial quadriceps tendon-femoral ligament (MQTFL) reconstruction). Results: In the static/anatomic technique, the patellar contact pressures at 0° and 30° were greater than in the native knee. As in a native knee, the contact pressures at 60°, 90°, and 120° were very low. The maximum MPFL graft stress at 0° and 30° was greater than in a native knee. However, the MPFL graft was loose at 60°, 90°, and 120°, meaning it had no tension. In the dynamic MPFLr using the AMT as a pulley, the patellar contact pressures were like those of a native knee throughout the entire range of motion. However, the maximum stress of the MPFL graft at 0° was less than that of a native ligament. Yet, the maximum MPFL graft stress was greater at 30° than in a native ligament. After 30° of flexion, the MPFL graft loosened, similarly to a native knee. In the dynamic MQTFL reconstruction, the maximum patellar contact pressure was slightly greater than in a normal knee. The maximum stress of the MPFL graft was much greater at 0° and 30° than that of a native MPFL. After 30° of flexion, the MQPFL graft loosened just as in the native knee. Conclusions: The patellar contact pressures after the dynamic MPFLr were like those of the native knee, whereas a static reconstruction resulted in greater pressures, potentially increasing the risk of patellofemoral osteoarthritis in the long term. Therefore, the dynamic MPFLr might be a safer option than a static reconstruction from a biomechanical perspective.

## 1. Introduction

Chronic lateral patellar instability (CLPI) is a common finding of knee surgeons in their daily clinical practice. Although its etiology is multifactorial, the most important structure in the development of this instability is medial patellofemoral ligament (MPFL) deficiency [1,2]. Over the last 10 years, MPFL reconstruction (MPFLr) has become a standard surgical technique for the treatment of CLPI, either as an isolated technique or combined with other surgical techniques [1,2,3,4,5,6]. Many surgical techniques using various types of grafts (autografts, allografts, or synthetic) and fixation techniques have so far been described. Based on the fixation technique used, there are two types of MPFLr, static and dynamic [1,7,8,9,10,11,12]. A static MPFLr, which involves an anatomic femoral bone attachment and a patellar bone attachment, is currently more common [1]. In the less used dynamic reconstruction, only one of the graft’s extremities is fixed to bone, while the other one is fixed to soft tissues. This type of reconstruction is therefore a less rigid reconstruction [7,8,9,10,11,12]. Static and dynamic reconstructions show MPFL isometry between 0° and 90° [9,13]. In a static/anatomic MPFLr, the graft is isometric in all the cases between 0° and 30° of knee flexion [13]. In 83% of cases, the graft is isometric from 0° to 60° [13]. Beyond 60° of knee flexion, the MPFL becomes progressively lax and isometry is lost [13]. Regarding isometry, a ligament is considered isometric when there is less than 5 mm of length change throughout the entire range of motion [14].

Both static and dynamic reconstruction techniques have been shown to yield satisfactory clinical results in the short and intermediate terms [1,7,8,12]. However, what is not known is whether one type of MPFLr is more likely than the other to lead to the development of patellofemoral osteoarthritis (PF OA) in the long term. We hypothesized that a static anatomic reconstruction might generate greater patellar contact pressure than a dynamic reconstruction and would therefore increase the risk of PF OA in the long term. Nevertheless, from a functional standpoint, a dynamic reconstruction would enable behavior that is more like that of a native MPFL than a static reconstruction. In a dynamic MPFLr, the fixation point gives a bit before the graft starts to stretch when the patella moves laterally. Therefore, the dynamic MPFLr allows for patellar contact pressures that are closer to those generated in a native knee. After the fixation of the MPFLr, it is important to verify that the patella can still be manually lateralized in full extension some 10 mm, to avoid any over-constraint, but with a firm endpoint. To avoid excessive tension on the graft when we perform an anatomic/static MPFLr, we have to fix the graft at 30° of knee flexion as it is at this angle that the distance between the femoral and patellar attachments points is greatest [13].

Cadaveric experiments have been conducted that show the achievement of physiological patellofemoral biomechanics, like that of the native knee with static MPFL reconstructions [15,16,17]. Cartilage degeneration after a static MPFLr has been related to graft overtension or an incorrect femoral attachment point [16,17]. Several biomechanical studies based on the finite element (FE) method have evaluated the MPFLr [18,19,20,21,22,23,24]. Previous studies were mainly based on a single FE model of the knee. Sanchis-Alfonso et al. [18] recently created a 3D parametric FE model of the patellofemoral joint (PFJ) that allowed for the evaluation of different types of MPFL reconstructions through the full range of motion of the knee. Additionally, the model is patient-specific. The objective of this paper was to evaluate the effect of different MPFL fixation techniques on patellar pressures compared with the native knee. High patellar pressures would be a risk factor for patellofemoral cartilage degeneration in the long term. This evaluation used a clinically-validated 3D parametric FE model of the PFJ [18].

## 2. Methods

Investigation performed at the Department of Mechanical Engineering, University of Zaragoza, Zaragoza, Spain.

### 2.1. Parametric FE Model of the PFJ

**Figure 1:**
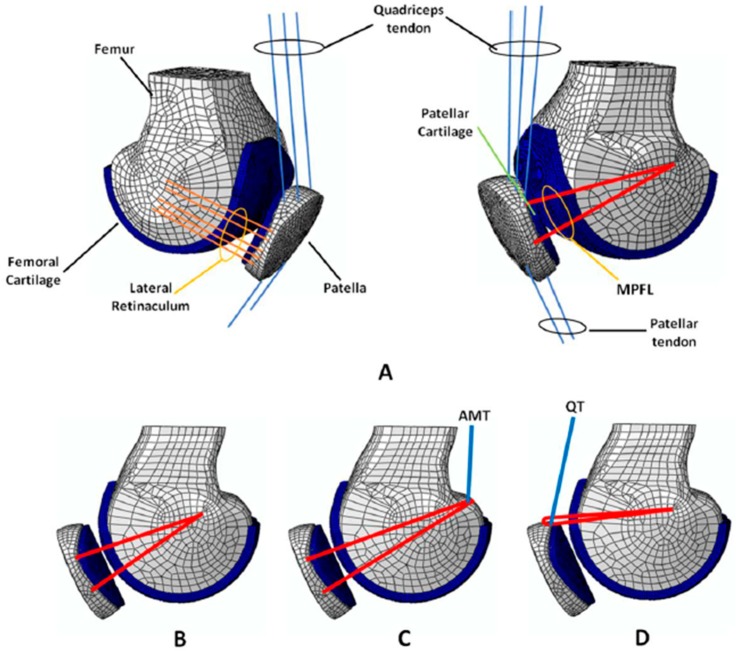
(**A**) Parametric geometry of the PFJ (femur, patella, and femoral and patella cartilages), (**B**) static/anatomic reconstruction, (**C**) dynamic reconstruction using AMT as the femoral fixation, and (**D**) dynamic reconstruction using the quadriceps tendon (QT) as one of the attachment points.

A parametric FE model of the PFJ was previously developed [18] based on the geometrical average of 24 knees acquired with a 64-detector multidetector computer tomography (CT) system (Philips, Best, The Netherlands) at the highest spatial resolution, without slice interpolation (0.255 × 0.255 × 0.672 mm^3^) [13]. The knee geometry was simplified (Figure 1). The main parts of the PFJ were the femoral condyle and patella bones, considered as rigid parts, and the femoral and patellar cartilages, considered as deformable components. A cartilage thickness of 3 mm was assumed [18,25]. Tendon and ligaments were also included to stabilize the patella and support the distribution of the patellar contact pressures better. The quadriceps tendon (QT), which consists of the vastus medialis (VM), vastus lateralis (VL), vastus intermedius (VI), and rectus femoris (RF) tendons, and the patellar tendon (PT) were modelled as a group of four and two truss elements, respectively (Figure 1), while the MPFL and the lateral retinaculum (LR) were defined as beam elements. The QT was oriented from the insertion site on the patella to the muscle origin or the most distal wrapping point on the femur, while the PT was oriented from the distal patella to the tibia [19,20]. The tendon and ligament properties were taken from previous studies and are summarized in Table 1 [21,26,27]. The cartilages were modelled with an elastic modulus of 10 MPa and a Poisson’s ratio of 0.45 [22,28,29]. A contact between both cartilage surfaces was defined with a friction coefficient of 0.02 [30]. Finally, the FE model of the PFJ consisted of around 30,700 nodes and 22,200 elements.

### 2.2. MPFLr Techniques

Three types of MPFLr were simulated: (a) static/anatomic reconstruction, meaning a reconstruction with a femoral anatomic fixation point and 2 patellar fixation points (Figure 1B); (b) dynamic reconstruction using the adductor magnus tendon (AMT) as the femoral fixation (Figure 1C); and (c) dynamic reconstruction using the quadriceps tendon as the attachment point (medial quadriceps tendon-femoral ligament (MQTFL) reconstruction) (Figure 1D). For the static reconstruction, the native ligament was simulated (intact knee). Two types of grafts were used for both the static and dynamic reconstructions using the AMT as a pulley. They were the semitendinosus autograft and gracilis autograft. These are the most frequently used grafts during MPFLr surgery. Two types of grafts were also used in the original MQTFL reconstruction (MQTFLr) technique described by Fulkerson and Edgar [12]. Those were the semitendinosus autograft and posterior tibial tendon allograft. For that reason, we performed two simulations during MQTFLr, using a semitendinosus autograft in one of them and a posterior tibial tendon allograft in the other. 

### 2.3. Simulation of the Different Surgical Techniques

The three surgical techniques were analyzed for 5 knee flexion positions: 0°, 30°, 60°, 90°, and 120°, as in a previous dynamic Computed Tomography (CT) scan study [13]. Table 2 summarizes the mean distance between the patella and femoral insertions points for the different MPFL reconstructions. Based on this data, the insertion nodes for each technique and the elongations suffered by the ligaments were determined. The reference position, where the ligaments did not experience any strain, was considered to be 40° of knee flexion. In some knee flexion positions, the distance between the femur and the patella insertion points was smaller than the reference distance (40°), which meant that the ligament was not experiencing any type of stress (Table 2, cases indicated by *). In other cases, the MPFL underwent an elongation (Table 2, cases indicated by +), which was simulated by applying a pretension force, ∆L × K, where ∆L is the length increment and K is the stiffness of the ligament (Table 1). LR lengths were assumed to be the same as the MPFL length to preserve the equilibrium on both sides of the joint. The average MPFL lengths were used in this part of the study to compare the performance of the static/dynamic reconstructions over the mean parametric FE model of the PFJ.

The parametric FE model and the simulations were carried out using the software Abaqus/CAE v.6.14 (Dassault Systèmes, Suresnes, France). Models were generated for each degree of knee flexion. First, the patella was brought closer to the femur to generate the contact. Then, the ligaments were incorporated. Subsequently, a previously calculated pretension force was applied to the ligaments little by little according to the length variation of the graft during the knee flexion (Table 2). It has been demonstrated that length variation of the graft during knee flexion differs in each type of MPFLr [13]. This way, the pressure of contact is generated. The initial contact pressures were removed to compare the different surgical techniques under the same conditions. Therefore, the results are presented as relative contact pressures. Maximum patellar contact pressures at each degree of knee flexion were evaluated. Maximum MPFL graft stress at each degree of knee flexion was then evaluated for the different positions of the knee.

## 3. Results

### 3.1. Static Anatomical Technique 

**Figure 2:**
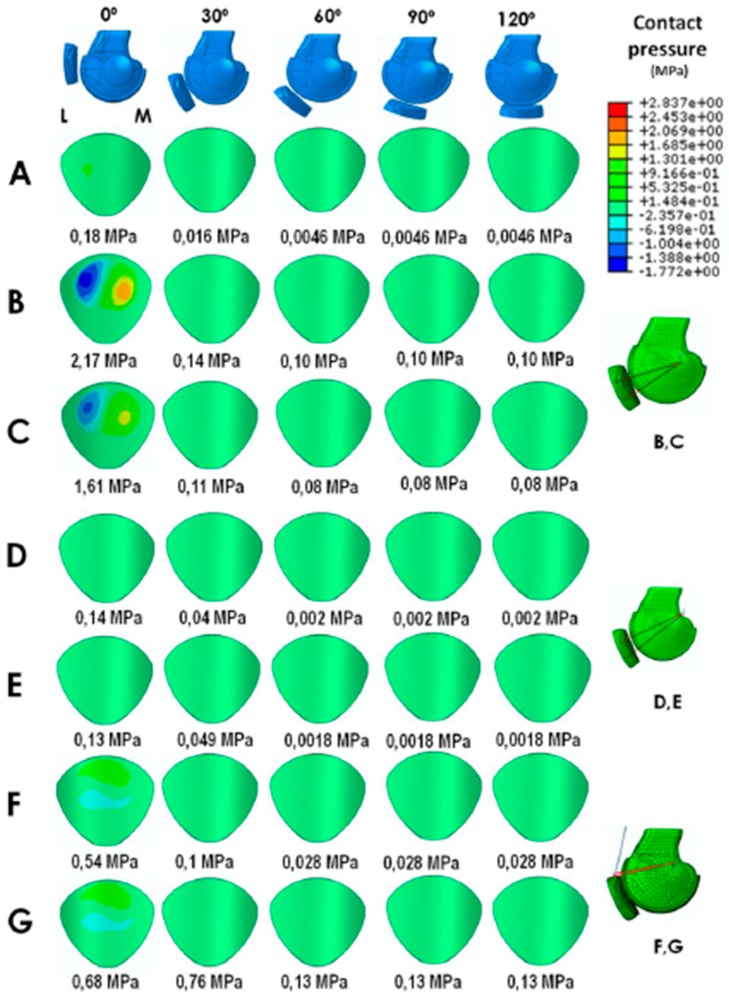
Contact pressure (MPa) on the patellar cartilage: (**A**) intact knee, (**B**) anatomic MPFLr with semitendinosus autograft, (**C**) anatomic MPFLr with gracilis autograft, (**D**) MPFLr with semitendinosus autograft using the AMT as a pulley, (**E**) MPFLr with gracilis autograft using the AMT as a pulley, (**F**) MQTFLr with semitendinosus autograft, and (**G**) MQTFLr with posterior tibial tendon allograft (M: Medial; L: Lateral).

With the static anatomical technique, the patellar contact pressures at 0° and 30° of knee flexion were greater than those of the native knee regardless of whether semitendinosus or gracilis autografts were used (Figure 2A–C). As in a native knee, the patellar contact pressures at 60°, 90°, and 120° were very low. The maximum patellar cartilage contact pressures are displayed in Figure 2B,C. The maximum MPFL graft stress at 0° and 30° was greater than in a native knee regardless of whether semitendinosus or gracilis autografts were used (Table 3). As in a native knee, at 60°, 90°, and 120° the MPFL graft was loose, meaning that it had no tension (Figure 2A–C). The MPFL and LR maximum stresses are displayed in Table 3.

### 3.2. Dynamic MPFLr Techniques 

In the dynamic MPFLr using the AMT as a pulley (Figure 2D–E), the patellar contact pressures were very similar to those of a native knee through the entire range of knee motion regardless of whether semitendinosus or gracilis autografts were used. The maximum patellar cartilage contact pressures are displayed in Figure 2D–E. The maximum MPFL graft stress was less at 0° than that of a native ligament when a gracilis autograft was used (Table 3). However, the maximum MPFL graft stress was greater at 30° than in a native ligament, regardless of whether a semitendinosus or gracilis autograft was used. After 30° of flexion, the MPFL graft loosened like in a native knee. The MPFL and LR maximum stresses are displayed in Table 3.

In the dynamic MQTFLr, using either a semitendinosus autograft or posterior tibial tendon allograft, the maximum patellar contact pressure was slightly greater than in a normal knee but lower than with the static anatomical technique (Figure 2F,G). The maximum patellar cartilage contact pressures are displayed in Figure 2F,G. The maximum stress of the MPFL graft using a semitendinosus autograft was much greater at 0° and 30° than that of a native MPFL (Table 3). With a posterior tibial tendon allograft, the maximum MPFL graft stress was greater than with a semitendinosus autograft. In all cases, after 30° of flexion, the MQPFL graft loosened like the native ligament. The MPFL and LR maximum stresses are displayed in Table 3.

## 4. Discussion

The most important finding of this study was that the patellar contact pressure from 0° to 30° of knee flexion, the range in which the patella is usually unstable [1,2], was lower in the dynamic reconstructions compared to the static anatomic reconstructions. In addition, the pressure was similar in dynamic reconstructions compared with an intact knee. This was consistent with our hypothesis. Our results, using a clinically validated FE parametric model [18], are different from those found by Rood et al. [31] in a controlled laboratory study using Tekscan pressure-sensitive films. According to these authors, the static MPFLr resulted in greater peak and mean pressures from 60° to 110° of flexion when compared to dynamic reconstructions. Moreover, these authors showed that the static MPFLr results in greater patellofemoral pressures and thus increases the risk of PF OA in the long term. On the other hand, the dynamic reconstruction results in more normal pressures. 

In the most commonly used dynamic MPFLr, the femoral attachment site uses the AMT as a pulley [7,8,10,11]. Some authors have tested the validity of this surgical technique, both clinically and radiologically, and found very satisfactory clinical results in the short term [7,8]. Fulkerson and Edgar [12] described the MQTFLr, another dynamic reconstruction technique. While the soft tissue technique using AMT is considered to be a non-anatomic technique, the soft tissue technique using the quadriceps tendon as the soft tissue fixation point is an anatomic technique as it reconstructs the MQTFL [32,33]. This technique also has good clinical results in the short term, like that of the static anatomic MPFL reconstructions with a patellar attachment point [12]. Moreover, this surgical technique essentially avoids the risk of patella fracture, which is a serious complication after MPFLr [34,35]. Both dynamic techniques restore the medial patella stabilizer, preventing lateral patella dislocation. Nevertheless, uncertainty currently exists relative to the long-term outcomes associated with these dynamic techniques, particularly with the development of PF OA. MPFLr evaluation by means of the FE parametric model, as done in the present study, is more sensitive than evaluations using clinical and radiological tests alone. It allows for the evaluation of patellar compression forces whose increment has been associated with the appearance of osteoarthritis in the tibiofemoral joint [36].

In the present study, we have shown that the dynamic technique using the AMT as a pulley with a gracilis tendon autograft (i.e., the most used graft) [8] not only does not increase the patellar contact pressure compared to an intact knee, but also shows a slightly lower resistance to rupture of the graft compared to a native ligament at 0° (Figure 2D–E, Table 3). In an ideal MPFLr, the graft should be more resistant than the native ligament to compensate for other instability predisposing factors [1]. Moreover, it is logical to think that if the reconstruction uses a graft with the same or lower maximum stress as the torn ligament, we are risking a new rupture. If the maximum stress is greater, then a repeat tear is less likely. Therefore, the aim is a stronger graft that will not tear again. However, this increment of the graft’s resistance should never be achieved by increasing the patellar contact pressure. Using a graft with the same or lower maximum stress as the ligament that has just tore could explain this technique’s failure when performed as an isolated MPFLr in patients with a severe trochlear dysplasia. Lind et al. [11] found that the outcomes after MPFLr with the gracilis tendon looped around the AMT insertion in pediatric patients were inferior to MPFLr using bony femoral fixation in adult patients (20% of the pediatric patients experienced redislocation within the first postoperative year compared with 5% of the adult patients). In this series, 20 out of 24 patients had some degree of trochlear dysplasia (10 cases were grade B and 10 cases were grade C or D, 42%) [11]. However, there was no correlation between high degree of trochlea dysplasia (grade C and D) and redislocations. Of the five redislocations, only two were seen in the 10 high-dysplasia knees. This uncorrected factor may have contributed to the high degree of redislocation observed in this series. Alm et al. [10] also found an elevated redislocation rate after MPFLr in children and adolescents when the adductor sling technique was used. The authors concluded that the adductor sling technique could only be recommended in the absence of additional patellofemoral maltracking, caused by an elevated tibial tuberosity-trochlear groove (TT-TG) distance (>15 mm), patella alta, or especially severe trochlear dysplasia. However, our clinical approach was to treat the associated predisposing factors for CLPI, and MPFLr was associated with realignment surgery in 56% of our cases. This approach could explain our satisfactory clinical results [8]. In our series of isolated MPFLr using the AMT as a pulley, the percentage of trochlear dysplasia grade C or D was only 8.5% (unpublished data). The fact that this type of reconstruction does not increase patellar contact pressure is very important because it indicates that it will not be predisposed to the development of a patellar chondropathy or PF OA in the long term. 

Our study showed that MQTFLr, from a biomechanical point of view, behaves like an anatomic static MPFLr (Figure 2F,G). The MQTFL graft was under tension during the first 30° of knee flexion, but it loosened after 30° and the already low patellar contact pressure decreased considerably after the first 30° (Table 3). MQTFLr significantly increased the resistance of the reconstruction without significantly increasing patellar contact pressure. This finding is very important because it indicates that this type of reconstruction is unlikely to contribute to the development of patellar chondropathy or PF OA in the long term. MQTFLr fulfills all the criteria for an ideal MPFLr from a biomechanical point of view. It combines a perfect balance between an optimal patellar contact pressure with the maximum graft stress, making a new tear less likely. 

Importantly, from a biomechanical standpoint, a dynamic reconstruction is better than a static one because it enables patellar contact pressure that is like that of an intact knee. To be able to definitely answer the question as to which reconstruction technique is better, the clinical results regarding the percentage of redislocations and functional results need to be considered. However, there are few clinical studies, and those that exist are of low quality with respect to the scientific evidence. We need well-designed, long-term prospective studies with many patients to answer these questions. 

### 4.1. Clinical Relevance

Our findings have important clinical relevance because they validated the use of MPFLr using the AMT as a pulley. The results are relevant not only for adults with a CLPI as a primary surgery, but also in certain situations, such as in revision surgeries with multiple bone tunnels or in children. In children, this method avoids injury to the distal femur growth plate and subsequent risk of developing a deformity of the knee [37]. Moreover, our study validated the use of MQTFLr not only as a primary surgery but also in the revision setting to avoid patellar problems.

Another interesting finding of our study was that the type of graft does matter, at least from a biomechanical point of view (Figure 2, Table 3). Numerous MPFLr surgical techniques, using autografts as well as allografts, have been described. From a clinical point of view, there seems to be no significant differences between the various types of grafts [38,39]. However, our FE parametric model study showed significant differences in terms of patellar contact pressure and the maximum MPFL graft stress. For example, the gracilis autograft has been recommended [8] in the MPFLr using the AMT as a pulley because the gracilis tendon appears to be long and strong enough to duplicate the MPFL function [40,41]. However, according to the results found using the FE method, the semitendinosus tendon has greater stress to failure relative to the gracilis, without significantly increasing the patellar contact pressure. In theory, a new tear is therefore less likely with a semitendinosus tendon autograft (Figure 2D,E, Table 3). 

### 4.2. Limitations

This study has several limitations. First, the PFJ was reconstructed from CT scans in which soft tissues cannot be clearly distinguished and cartilage thickness was estimated by taking a fixed measure into account. The inclusion of magnetic resonance (MR) data from the same patients and the use of image registration techniques to merge MR and CT data would not only allow for measuring cartilage thickness accurately but also for patient-specific matrix properties, such as the T1 or T2 relaxation time, related to proteoglycan and collagen matrix integrity, respectively. Various material models have been developed to describe the mechanical behaviour of articular cartilage [42,43,44,45,46]. Due to computational costs and time-consuming nature associated with 3D FE modelling of more complex models, cartilage has been considered to be a homogenous, isotropic, and linearly elastic material, and interstitial fluid flow has been neglected. Second, ligament material properties were taken from the literature [21,26,27]. In the future, patient-specific material properties could be considered. Third, greater patellar contact pressure is an important risk factor for developing PF OA in the long term. However, the pressure values that will produce symptomatic PF OA are unknown. Segal et al. [36] observed that a threshold of 3.42 to 3.61 MPa had 73.3% sensitivity, with specificity from 46.7% to 66.7%, regarding the prediction of symptomatic knee osteoarthritis. Obviously, these values cannot be extrapolated to the PFJ, which is the joint with the thickest cartilage in the human body. However, it is logical to think that the pressures causing a symptomatic PF OA would be greater. Several experimental studies have included a certain amount of the quadriceps force applied to the patella [21,31] to determine the patella femoral spatial relationship and contact pressure. Our model did not incorporate this force. It was not necessary because the model itself has stability and because we were only comparing three techniques.

## 5. Conclusions

The patellar contact pressures after dynamic MPFLr were like those of the native knee, whereas static reconstruction resulted in greater pressures and, thus, could eventually increase the risk of PF OA in the long term. Therefore, dynamic MPFLr might be a safer option than static reconstruction from a biomechanical point of view. We need long-term clinical studies with both dynamic and static techniques to corroborate the conclusions that were obtained with our biomechanical study using an FE parametric model.

## Figures and Tables

**Table 1:** Material properties considered for the FEM (Finite Element Model) simulation (from references [21,26,27]).

Material Properties		
	Stiffness (N/mm)	Poisson Ratio
Quadriceps Tendon (QT)	1350	0.3
Patellar Tendon (PT)	2000	0.3
Lateral Retinaculum (LR)	2	0.3
Native Medial Patellofemoral Ligament (MPFL)	12	0.3
MPFL Reconstruction (Semitendinosus Autograft)	100	0.3
MPFL Reconstruction (Gracilis Autograft)	80	0.3
MQTFL Reconstruction (Semitendinosus Autograft)	100	0.3
MQTFL Reconstruction (Posterior Tibial Allograft)	513	0.3

**Table 2:** Intact MPFL and different types of MPFL reconstruction (MPFLr) data (* ligaments under no tension, + ligaments under tension).

	Anatomic MPFLr (STATIC)	MPFLr Using the AMT as a Pulley (Superior Bundle) (DYNAMIC)	MPFLr Using the AMT as a Pulley (Inferior Bundle) (DYNAMIC)	MQFTLr (DYNAMIC)
Flexion Angle (°)	Length (mm)	Length (mm)	Length (mm)	Length (mm)
0	60.2 +	61.1 +	58.3 +	65 +
30	57.9 +	60.9 +	60.1 +	63 +
40	57.7	60.8	60.8	62.7
60	57.3 *	60.7 *	62.1 *	62 *
90	55.6 *	60.4 *	62.2 *	62 *
120	50.7 *	55.8 *	57 *	62 *

AMT = Adductor Magnus Tendon.

**Table 3:** MPFL and LR maximum stress (MPa) values for each case analyzed.

Ligament Status	Flexion Angle (°)	Maximum MPFL Stress (MPa)	Maximum LR Stress (MPa)
INTACT MPFL	0	8.85	1.52
	30	0.78	0.15
ANATOMIC MPFLr Semitendinosus autograft	0	74.72	1.51
	30	6.55	0.14
ANATOMIC MPFLr Gracilis autograft	0	58.78	1.51
	30	5.12	0.14
MPFLr with AMT as a Pulley Semitendinosus autograft	0	7.11	0.15
	30	2.53	0.05
MPFLr with AMT as a Pulley Gracilis autograft	0	6.35	0.15
	30	2.10	0.05
MQTFLr Semitendinosus autograft	0	66.70	1.42
	30	9.36	0.23
MQTFLr Posterior Tibial allograft	0	100.80	1.42
	30	49.76	0.23
